# Influence of Admission Blood Glucose in Predicting Outcome in Patients With Spontaneous Intracerebral Hematoma

**DOI:** 10.3389/fneur.2018.00725

**Published:** 2018-08-28

**Authors:** Lakshman I. Kongwad, Ajay Hegde, Girish Menon, Rajesh Nair

**Affiliations:** Department of Neurosurgery, Kasturba Medical College, Manipal Academy of Higher Education, Manipal, India

**Keywords:** intracerebral hemorrhage, mRS, stroke, admission blood glucose, stroke outcome

## Abstract

**Background and Aims:** Hyperglycemia or elevated blood glucose levels have been associated with poor outcomes in patients with ischemic stroke yet control of hyperglycemia has not resulted in good outcomes. High admission blood glucose (ABG) values have been mitigated by other poor prognosticators like large hematoma volume, intraventricular extension (IVE) of hematoma and poor GCS. The aim of this study was to evaluate the effects of blood glucose levels at admission, on mortality and functional outcomes at discharge and 3 months follow up.

**Methods:** This was a retrospective observational study conducted at a tertiary care. Patients with spontaneous SICH were enrolled from a prospective SICH register maintained at our hospital. Blood glucose values were recorded on admission. Patients with traumatic hematomas, vascular malformations, aneurysms, and coagulation abnormalities were excluded from our study.

**Results:** A total of 510 patients were included in the study. We dichotomised our cohort into two groups, group A with ABG>160 mg/dl and group B with ABG<160 mg/dl. Mean blood glucose levels in these two groups were 220.73 mg/dl and 124.37 mg/dl respectively, with group A having twice the mortality. mRS at discharge and 3 months was better in Group B (*p* ≤ 0.001) as compared to Group A. Age, GCS, volume of hematoma, ABG, IVE and Hydrocephalus were significant predictors of mortality and poor outcome on univariate analysis with a *p* < 0.05. The relationship between ABG and mortality (*P* = 0.249, 95% CI 0.948–1.006) and outcome (*P* = 0.538, 95% CI 0.997–1.005) failed to reach statistical significance on multivariate logistic regression. Age, Volume of hematoma and GCS were stronger predictors of mortality and morbidity.

**Conclusion:** Admission blood glucose levels was not an independent predictor of mortality in our study when adjusted with age, GCS, and hematoma volume. The effect of high ABG on SICH outcome is probably multifactorial and warrants further research.

## Introduction

Spontaneous Intracerebral Hemorrhage (SICH) accounts for 15 to 20% (1, 2) of all strokes encountered in a tertiary care hospital and is associated with higher mortality and morbidity when compared with ischemic strokes(3, 4). Stress hyperglycemia is associated with a high risk of mortality after both stroke (5) and myocardial infarction (6). Hyperglycemia at the time of stroke has been associated with poor prognosis in ischemic stroke (7, 8). However in ICH, literature shows conflicting results with few studies showing that high blood glucose could have a biologically plausible association with poor outcomes and death (9–17).

High blood glucose level is related to brain edema and neuronal apoptosis in ICH (18). Hyperglycemia is also reported to promote blood-brain barrier destruction via downregulation of Aquaporin-4 channels (19). Studies have shown that patients with elevated admission blood glucose (ABG) levels were associated with an increased incidence of cerebral complications and more widespread bleeding, re-bleeding, brain oedema, and poor functional outcome at the time of discharge (20). However the association is still controversial whether admission hyperglycemia independently affects outcomes or is associated with other prognostically poor features of stroke (8, 12, 21–23).

In this study we aim (a) to evaluate if ABG levels can be an independent predictor of mortality and outcome and (b) to correlate the values of blood glucose with other predictors of mortality and outcome in our cohort.

## Materials and methods

This was a retrospective observational study conducted at Kasturba Hospital Manipal, a tertiary care center in the small coastal town in of Udupi, Karnataka, India. All consecutive patients with spontaneous SICH from February 2015 to July 2017 were enrolled in our study from a prospective SICH register maintained at our hospital. Data pertaining to age, sex, hypertension, diabetes, admission systolic, and diastolic blood pressures, admission glucose level, Glasgow Coma Scale (GCS) score, volume of hematoma, presence of intraventricular hemorrhage, hydrocephalus were collected from the database. Patients with traumatic hematomas, vascular malformations, aneurysms, and coagulation abnormalities were excluded from our study. Hematoma volume was calculated by the (abc)/2 method (24, 25) on 5 mm slices of non-contrast CT on admission. Admission blood glucose values were recorded from venous sample by Hexokinase method on admission. Modified Rankin scale (mRS) was used to assess outcome at discharge and at 3 months follow up. Telephone assessment mRS, shown to have good inter-rater reliability was used for patients who did not present to the clinic at 3 months (26). Patients with mRS of 0–3 were classified as good outcome and 4–6 as poor outcome. Mortality was tabulated with the mRS available at 90 days.

### Statistical analysis

The primary outcome determinants considered were mortality and outcome, as measured using mRS at discharge and 3 months. Analysis was performed using IBM SPSS V24 and R V 3.4.1 for Macintosh. Descriptive statistics included mean, median and standard deviation. Continuous variables were analyzed using Student *t*-test for normally distributed data and Mann-Whitney test for skewed data. Categorical variables were analyzed with Chi Square test. Pearson correlation coefficient was used to determine correlation between continuous variables. At multivariate level, logistic regression analysis was used to determine variables associated with mortality and poor functional outcome. Variables with *p* < 0.05 at univariate level were included in multivariate modeling. Outcomes were reported as odds ratios (OR) with 95% confidence interval (CI). ROC curve analysis was performed for blood glucose value to determine cut-off levels that predicted death. A *p*-value of < 0.05 was considered significant.

## Results

A total of 510 patients were analyzed in our study. The mean age of our study group was 58.39 years with a range of 23–93 years. One hundred forty-six patients (28.62%) were below the age of 50 years with predominantly males (M:F::363:147). Five patients were excluded from our analysis as ABG was not available.

**Figure 1:**
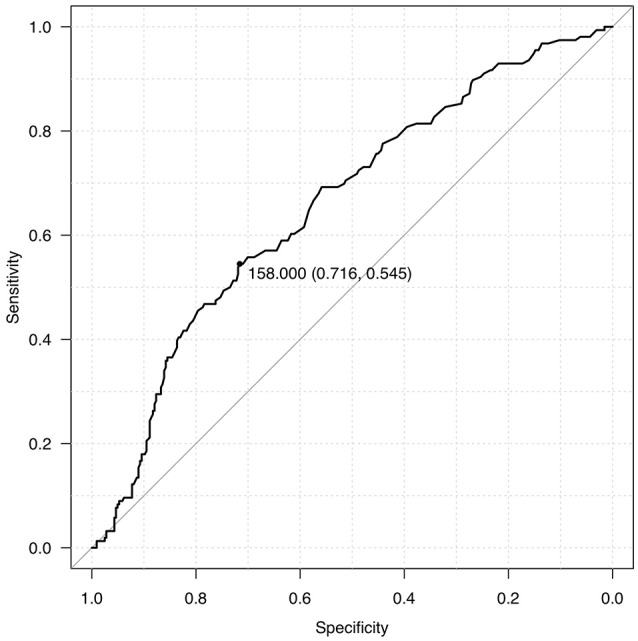
ROC curve for blood glucose levels.

We dichotomized our cohort into 2 groups, Group A and Group B, based on admission blood glucose. ROC curve was used to determine the midpoint of the curve (Figure 1) and patients with ABG >160 mg/dl were categorized as Group A and ABG<160 mg/dl as Group B (Table 1). Though the mean age seemed to be identical in both the cohorts there was a striking male preponderance (Group A M:F; 126:51, Group B M:F; 234:94). Yet another paramount finding noted was that of a lower ABG in diabetics as compared to non-diabetics (36 in Group A as compared to 103 in Group B).

**Table 1:** Demographic, clinical, radiological, and outcome of our dichotomized cohort.

**Parameter**	**GRBS>160 (*n* = 177)**	**GRBS<160 (*n* = 328)**	***p***
**DEMOGRAPHICS AND RISK FACTORS**			
Age (years)	58.37 ± 12.99	58.42 ± 12.32	0.96
Male	126 (35%)	234 (65%)	
Female	51 (35.2%)	94 (64.8%)	
Hypertension	106 (39.8%)	160 (60.2%)	0.011
Duration in years	5.7 ± 4.81	4.7 ± 4.7	
Diabetics	36 (25.9%)	103 (74.1%)	0.005
Duration in years	8.9 ± 5.98	5.39 ± 4.16	
Smoking	47 (48.4%)	50 (51.6%)	
Alcohol	69 (37.5%)	115 (62.5%)	0.244
**CLINICAL FEATURES**			
Heart rate (bpm)	84.7 ± 18.24	82 ± 16.38	
Systolic BP (mmHg)	184.29 ± 29.51	171.34 ± 27.56	<0.001
Diastolic BP (mmHg)	103.33 ± 13.76	99.1 ± 12.83	0.001
GCS (median)	11	12	<0.001
GCS < 8	67 (54.9%)	55 (45.1%)	<0.001
ICH score (median)	2	1	<0.001
Blood glucose (mg/dl)	220.73 ± 57.23	124.37 ± 20.05	<0.001
Supratentorial hemorrhage	146	298	
Volume (ml)	31.54 ± 23.12	19.55 ± 13.77	<0.001
>30 ml	72	61	<0.001
Infratentorial hemorrhage	31	30	
IVE	109 (47.6%)	120 (52.4%)	<0.001
Hydrocephalus	57 (45.6%)	68 (54.4%)	0.003
Hematoma growth	9 (39.1%)	14 (60.9%)	0.36
EVD	17 (47.2%)	19 (52.8%)	0.081
Surgery	45 (53.6%)	39 (46.4%)	<0.001
**OUTCOME**			
MRS at discharge	5	4	<0.001
MRS at 3 months	5	3	<0.001
MRS 0–3	63 (26.5%)	174 (73.5%)	<0.001
MRS 4–6	109 (42.4%)	147 (57.5%)	
**DEATH**			
In Hospital	31 (56.3%)	24 (43.7%)	
3 months	81 (50.9%)	78 (49.1%)	<0.001

The mean systolic blood pressures were 184.29 and 171.34 in the two groups respectively and diastolic pressures were 103.33 and 99.1 in Group A and Group B, respectively.

Median Glasgow Coma Scale was 11 in Group A and 12 in Group B (*P* ≤ 0.001). Median ICH score was 2 in Group A and 1 in Group B. Mean ABG value was 220.73 ± 57.23 mg/dl (160–407 mg/dl) in Group A and 124.37 ± 20.0 mg/dl (42–159 mg/dl) in Group B.

Confounding factors such as hematoma volume, presence of intraventricular extension, anatomical location, and GCS on arrival also seemed to affect the outcome of patients with SICH. In Group A, 141 patients had supratentorial hematomas with a mean volume of 31.54 ± 23.12 ml (5–110 ml), and 72 patients with a volume >30 ml. Thirty-one patients had infratentorial hematomas. One hundred nine patients had intraventricular extension (IVE), with 57 having imaging suggestive of hydrocephalus. Sixteen patients among these required an EVD and 45 patients underwent surgical evacuation of hematoma.

In Group B, 283 patients had supratentorial hematomas, with a mean volume of 19.5 ± 13.77 ml (3–70 ml) and 61 patients had a hematoma volume >30 ml. Thirty patients had infratentorial hematomas. One hundred twenty patients had intraventricular extension, with 68 patients having hydrocephalus on imaging. Nineteen patients had an EVD inserted and 39 patients underwent surgical evacuation of hematoma.

In terms of mean hematoma volumes, patients in Group A had mean hematoma volumes almost twice that of group B and 64% of patients in group A presented with IVE in comparison to 36% in group B. Pearson's correlation of ABG with Volume of hematoma, GCS and IVE showed a strong correlation with *p* < 0.001 and *R*-values of 0.249, −0.267, and 0.261, respectively (Table 2).

**Table 2:** Pearson's Correlation coefficient for blood glucose vs. parameters influencing mortality.

**Parameter**	***R***	***P***
Volume of hematoma	0.249	<0.001
GCS	−0.267	<0.001
IVE	0.261	<0.001

### Outcome assessment

Outcome mRS at 3 months was unavailable for 12 patients as they were lost to follow up. Telephonic mRS was obtained for 206 patients. Median mRS among the patients in Group A was 5, with 63 (36.6%) patients having an mRS of 0–3 and 109 (63.4%) with an mRS of 4–6. Thirty-one patients died in the hospital and another 50 within 90 days. In Group B, the median mRS was 4 at discharge and 3 at 90 days with 174 (54.2%) patients having good outcome and 147 (45.8%) with poor outcome at 3 months. Twenty-four patients died in the hospital and 54 in the first 3 months. Age, GCS, volume of hematoma, admission blood glucose, IVE and Hydrocephalus were significant predictors of mortality and poor outcome on univariate analysis with a *p* < 0.05 (Table 3).

**Table 3:** Univariate analysis for mortality and outcome.

**Parameter**	**Mortality**				**Poor outcome**			
	**Alive**	**Dead**	***P***	**95% CI**	**Good**	**Bad**	***P***	**95% CI**
Age (mean)	56.95 ± 11.84	61.54 ± 31.29	<0.001	2.285 to 6.908	56.63 ± 11.87	60.12 ± 12.87	0.002	−5.673 to −1.299
GCS	12.11 ± 2.68	8.09 ± 3.67	<0.001	−4.587 to −3.445	9.1 ± 3.61	12.64 ± 2.46	<0.001	2.988 to 4.088
Volume	17.59 ± 13.23	31.82 ± 22.18	<0.001	11.095 to 17.359	15 ± 11.32	28.89 ± 20.25	<0.001	−16.82 to −10.96
Blood glucose	150.17 ± 58.48	177.20 ± 59.29	<0.001	15.914 to 38.147	147.04 ± 55.41	169.86 ± 62.09	<0.001	−33.267 to −12.376
IVE	127 (55%)	104 (45%)	<0.001		81 (35.1%)	150 (64.9%)	<0.001	
Hydrocephalus	60 (47.2%)	67 (52.8%)	<0.001		40 (31.5%)	87 (68.5%)	<0.001	

### Multivariate logistic regression

Multivariate Logistic regression was performed to determine the relationship between the Age, GCS, Volume of hematoma, ABG, IVE, and presence of hydrocephalus with mortality and poor outcome (Table 4). It failed to demonstrate association between blood glucose and mortality (*P* = 0.249, 95% CI 0.948–1.006) and outcome (*P* = 0.538, 95% CI 0.997–1.005). Age, Volume of hematoma and GCS were stronger predictors of mortality and morbidity (Table 4).

**Table 4:** Multiple logistic regression for mortality and poor outcome.

**Parameter**	**Mortality**			**Poor Outcome**		
	**Odds ratio**	***P***	**95% CI**	**Odds ratio**	***P***	**95% CI**
Age	1.06	<0.001	1.030–1.083	1.050	<0.001	1.030–1.070
GCS	0.721	<0.001	0.663–0.783	0.730	<0.001	0.661–0.793
Volume	1.029	<0.001	1.013–1.045	1.051	<0.001	1.031–1.071
Blood glucose	1.002	0.249	0.948–1.006	1.001	0.538	0.997–1.005
IVE	0.973	0.923	0.556–1.702	0.694	0.166	0.413–1.164
Hydrocephalus	0.650	0.172	0.351–1.206	0.915	0.781	0.489–1.713

## Discussion

Diabetes and high blood glucose are well-known risk factors for ischemic stroke (5, 27–29). While a number of ischemic studies showed worse outcomes with hyperglycaemia (7) others have shown protective effect of hyperglycaemia in small vessel disease (30). A number trials attempting to control hyperglycaemia in ischemic stroke patients have been negative (7, 31).

High blood glucose values have shown a strong association with higher mortality and morbidity in multiple cohorts, independent to the presence of Diabetes Mellitus (9, 14, 15, 17, 32, 33), while some others (8, 12, 23) have shown contrasting reports like ours. Two Meta-analysis have concluded that hyperglycaemia increases both short term and long term mortality in intracerebral hemorrhage (15, 16). However these included studies with subarachnoid hemorrhage also. A recent meta-analysis by Zheng et al. (34) in 2018, concluded that hyperglycemia was associated with poor functional outcome in patients with ICH. The wide range of blood glucose levels and heterogeneity among studies could be a reason for bias in these meta-analyses. Random blood glucose has not been a predictor of mortality in Indian ICH studies (21, 22). However, the pool of available evidence pertaining to blood glucose variability and ICH is still limited (35–37).

Several animal studies have also identified an evident association between hyperglycemia and perihematomal neuronal apoptosis in rat models (38). They also predicted that high blood glucose level was strongly associated with increased neurologic injury and decreased autophagy (39). This was attributed to a direct causal effect of hyperglycemia, which enhances the synthesis of oxygen free radicals (super oxide) and also responsible for the down regulation of super-oxide dismutase. Superoxide, thus produced, induces the synthesis of tissue plasminogen activators thereby de-stabilizing the clot and causing hematoma expansion in ICH models via plasma kallikrein (40, 41). Decline of blood glucose within 72 h of ictus has also been correlated with reduction in hematoma expansion and poor clinical outcome (42).

In a multivariate analysis, ABG >8 mmol/l appeared to be a significant independent predictor of death within 2 days of stroke onset (43). In the 127 patients who died in a study by Fogelholm et al. mean blood glucose values were 9.1 mmo/L in comparison to 6.8 mmol/L among survivors with a *p* < 0.01 (11). Kimura et al. in their study of 100 patients also found that higher blood glucose values on presentation was directly associated with a higher mortality (glucose: death, 205 mg/dl vs. survival, 131 mg/dl, (*p* < 0.0001) (20). A similar observation was noticed in our study too with the mean blood glucose among patients who died in being 177.20 mg/dl compared to 150.17 in those who survived for 90 days (*P* < 0.001, 95% CI = 15.914–38.147) on univariate analysis.

Lee et al. (12) conducted a prospective study across 33 centers in Korea and concluded that admission glucose was an independent risk factor for early mortality in patients with diabetes and it was an independent risk factor for long term mortality (*p* = 0.09) in non-diabetics. Long term mortality in diabetics remained unaffected. Another study conducted by Passero et al. (10) in Italy, projected that elevated admission glucose in patients with ICH was an independent predictor of 3 day and 3 month mortality in non-diabetic, non-comatose patients after supratentorial ICH. In a large retrospective study from the China National Stroke Registry comprising of 2,951 patients, Sun et al. (17) concluded that elevated admission glucose level is an independent predictor of 90-day poor outcome, the prognostic value of which is greater in non-diabetics than diabetics. The likely cause for this difference was probably stress hyperglycemia, insulin resistance, undiagnosed diabetes and undertreatment. Our study too found diabetics to have a better control of glucose levels (*p* = 0.005).

The present study shows that elevated blood glucose at the time of SICH was associated with a more severe form of ICH, accounting for increased mortality and morbidity. Hyperglycaemia in the setting of acute neurological injury is attributed to a catecholamine surge and the generalized stress response (8). It is proposed that high blood glucose at admission contributes to poor outcome, due to exacerbation of cerebral edema and cerebral damage (44). Though several animal studies have shown that ICH promotes vasogenic oedema and perihaematomal neuronal death, some authors have noticed that elevated ABG could only reflect the severity of the ICH as a stress reaction to a serious brain injury (39).

**Figure 2:**
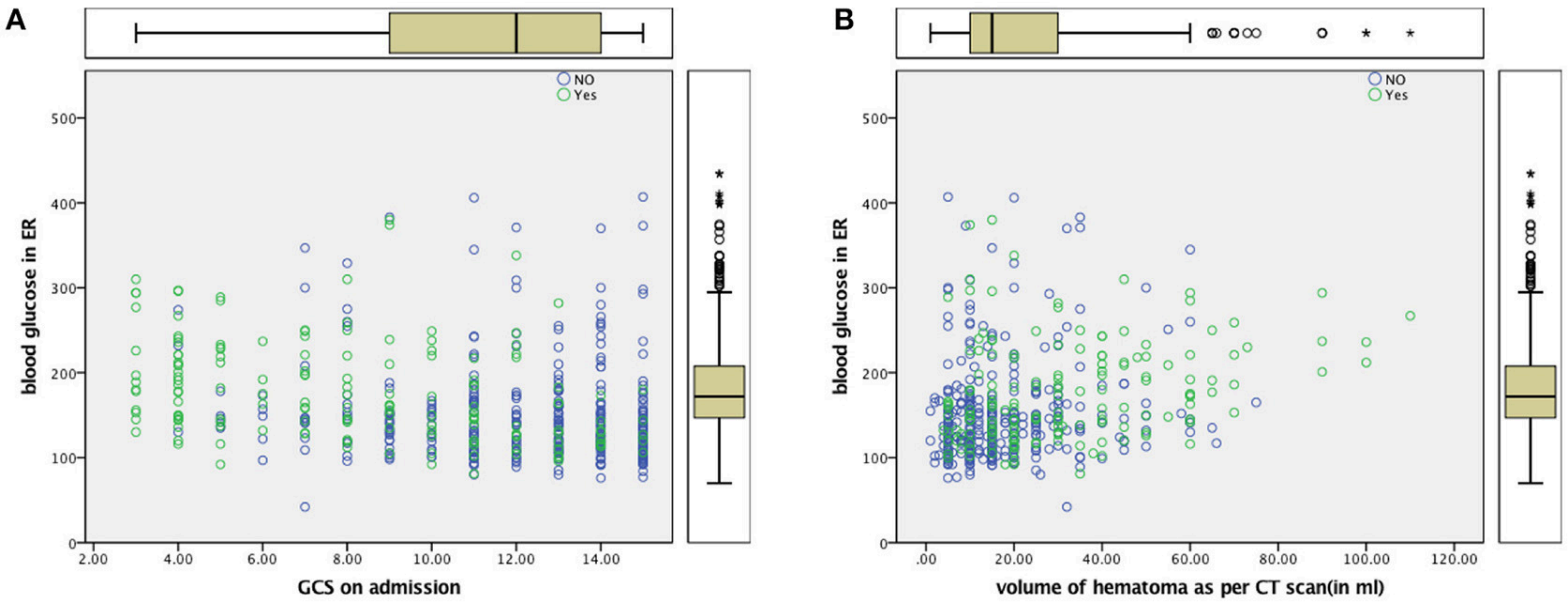
**(A)** Scatter Plot for ABG and GCS **(B)** Scatter Plot for ABG and Volume of Hematoma Dead—Yes (Green), Alive—No (Blue).

Multivariate logistic regression failed to uphold ABG as an independent predictor of mortality in our study when adjusted for age, GCS, and hematoma volume. The loss of significance of blood glucose in multivariate models may be due to confounding and possible statistical interaction by GCS, Age, and Hematoma volume. These are well known, established independent factors of poor outcome and mortality in ICH [Figure 2; (2, 45–48)]. This could be attributed to a stress response (49) following ICH as was seen in the Fogelholm study (11) of Finland and the Italian study of Passero et al. (10). Ntaios et al. (35) have pointed out in the realm of ischemic strokes that the relationship between admission glucose and outcomes may be J-shaped and best modeled by fractional polynomials. In light of complex relationships between glucose and outcomes and heterogeneity in neurological conditions, Kent et al. (50) questioned whether underlying assumptions of multivariate regression modeling are met.

Intensive blood glucose lowering with the use of insulin has failed to provide favorable outcomes in patients with ICH (51, 52) SAH (53) and ischemic stroke (54). A tight glycemic control may result in brain energy crisis which is associated with a high rate of mortality (55). When effective lowering of blood glucose in these studies have failed to improve outcome, it is unlikely that elevated blood glucose is the cause or effect of mortality in the different proposed mechanisms of increasing brain oedema and perihematoma cell death (18). It is probable that high ABG was associated with variables signifying the severity of stroke, such as blood pressure, hematoma volume, midline shift of the cerebral structures, intraventricular extension, and poor sensorium. Patients in Group A had larger volume bleeds, and patients with poorer GCS. This translated into far more deaths among patients in Group A and poorer clinical outcome thereafter. Whether the adverse effect of high blood glucose levels manifests through larger clot volumes needs to be further explored with newer technologies of continuous blood glucose monitoring (56, 57).

## Conclusion

Admission blood glucose levels was not an independent predictor of mortality in our study when adjusted with age, GCS and hematoma volume. GCS and volume of hematoma were better predictors of mortality at 90 days while volume of hematoma and age were better predictors of functional outcome. Catastrophic SICH is associated with high ABG, which appears to be a stress response to the severity of bleeding. The effect of high blood glucose levels on SICH outcome is probably multifactorial and warrant further research.

## Ethics statement

This study was carried out in accordance and approval of the ethics committee of Manipal Academy of Higher Education vide approval no IEC 209/2015.

## Author contributions

LK was the primary investigator involved in write up of the article. AH was involved in data collection, follow up of patients and tabulation of results. GM and RN were involved in review of article.

### Conflict of interest statement

The authors declare that the research was conducted in the absence of any commercial or financial relationships that could be construed as a potential conflict of interest.
